# Transcriptome analysis of sweet orange trees infected with ‘*Candidatus* Liberibacter asiaticus’ and two strains of *Citrus Tristeza Virus*

**DOI:** 10.1186/s12864-016-2663-9

**Published:** 2016-05-11

**Authors:** Shimin Fu, Jonathan Shao, Changyong Zhou, John S. Hartung

**Affiliations:** College of Plant Protection/Citrus Research Institute, Southwest University, Chongqing, China; Molecular Plant Pathology Laboratory, United States Department of Agriculture-Agricultural Research Service, Beltsville, MD USA; Lingnan Normal University, Zhanjian, China

**Keywords:** Huanglongbing, Gfold, Host-pathogen interactions

## Abstract

**Background:**

Huanglongbing (HLB) and tristeza, are diseases of citrus caused by a member of the α-proteobacteria, ‘*Candidatus* Liberibacter asiaticus’ (CaLas), and *Citrus tristeza virus* (CTV) respectively. HLB is a devastating disease, but CTV strains vary from very severe to very mild. Both CaLas and CTV are phloem-restricted. The CaLas-B232 strain and CTV-B6 cause a wide range of severe and similar symptoms. The mild strain CTV-B2 doesn’t induce significant symptoms or damage to plants.

**Results:**

Transcriptome profiles obtained through RNA-seq revealed 611, 404 and 285 differentially expressed transcripts (DETs) after infection with CaLas-B232, CTV-B6 and CTV-B2. These DETs were components of a wide range of pathways involved in circadian rhythm, cell wall modification and cell organization, as well as transcription factors, transport, hormone response and secondary metabolism, signaling and stress response. The number of transcripts that responded to both CTV-B6 and CaLas-B232 was much larger than the number of transcripts that responded to both strains of CTV or to both CTV-B2 and CaLas-B232. A total of 38 genes were assayed by RT-qPCR and the correlation coefficients between Gfold and RT-qPCR were 0.82, 0.69, 0.81 for sweet orange plants infected with CTV-B2, CTV-B6 and CaLas-B232, respectively.

**Conclusions:**

The number and composition of DETs reflected the complexity of symptoms caused by the pathogens in established infections, although the leaf tissues sampled were asymptomatic. There were greater similarities between the sweet orange in response to CTV-B6 and CaLas-B232 than between the two CTV strains, reflecting the similar physiological changes caused by both CTV-B6 and CaLas-B232. The circadian rhythm system of plants was perturbed by all three pathogens, especially by CTV-B6, and the ion balance was also disrupted by all three pathogens, especially by CaLas-B232. Defense responses related to cell wall modification, transcriptional regulation, hormones, secondary metabolites, kinases and stress were activated by all three pathogens but with different patterns. The transcriptome profiles of *Citrus sinensis* identified host genes whose expression is affected by the presence of a pathogen in the phloem without producing symptoms (CTV-B2), and host genes whose expression leads to induction of symptoms in the plant (CTV-B6, CaLas-B232).

**Electronic supplementary material:**

The online version of this article (doi:10.1186/s12864-016-2663-9) contains supplementary material, which is available to authorized users.

## Background

Citrus is grown in more than one hundred countries and is one of the most important commercial fruit crops in the world. Citrus is affected by different diseases and different geographic areas have distinct disease problems. Tristeza and huanglongbing (HLB) are two of the most destructive and widely distributed diseases [1]. Tristeza is caused by *Citrus tristeza virus* (CTV), a member of the *Closteroviridae* that is aphid-transmitted and phloem-restricted, with a single-stranded and positive sense RNA genome. CTV can be classified into mild and severe types based on the severity of symptoms produced on citrus indicators. Mild CTV strains cause only mild or no symptoms on indicators and usually bring about no economic loss. Severe CTV strains induce different disease syndromes, including decline and death of sweet orange on sour orange rootstocks, stem pitting (SP) of sweet orange and grapefruit scions, seedling yellows (SY) in sour orange, and vein clearing in ‘Mexican’ lime seedlings [2, 3]. HLB is associated with a member of the α-subdivision of the proteobacteria: ‘*Candidatus* Liberibacter asiaticus’ (CaLas), which grows only in the phloem of infected plants and the hemocoel and salivary glands of infected insect vectors. Because CaLas has not been grown in pure culture and Koch’s postulates have not been completed, it has ‘*Candidatus*’ status in the accepted nomenclature [4, 5]. HLB affected trees may produce new shoots that are uniformly yellow but mature to present blotchy mottle symptoms, usually with symptoms of zinc deficiency. Fruit are misshaped with reduced quality and quantity, abnormal coloration and aborted seeds. HLB is considered to be one of the most serious plant diseases in the world and there are no resistant varieties. Growers attempt to maintain yield in the presence of the disease with nutritional supplementation [6], and rigorous control of the citrus psyllid vector even after trees in a grove have already been infected by CaLas [7]. Benefits in terms of yield of these treatments have been reported [6, 7].

An understanding of host responses to pathogen infection is essential to clarify the mechanisms of plant-microbe interactions and to develop novel strategies for therapy and detection. Pathogens may activate plant defense responses, such as fortification of cell walls, the production of reactive oxygen species, phytoalexins and signaling molecules and the synthesis of pathogenesis-related proteins [8]. Transcriptome studies of the host can provide valuable information on such defense responses and may lead to new plant protection strategies. Transcriptome analyses of citrus plants in response to CTV and CaLas have been reported in separate studies [9–12]. Many host genes and related metabolic pathways and processes were affected by CaLas [10] and severe strain CTV-T305 compared to mild strain CTV-T385 [12].

Both CTV and CaLas are phloem-restricted pathogens and can occur separately or in combination on an individual tree. Mild CTV strains don’t cause damage to citrus hosts, while severe CTV strains and CaLas have overlapping symptom patterns. Therefore, we present a comparison of transcriptome profiles of citrus plants infected with a mild CTV strain, severe CTV strain and CaLas. We hypothesized that many host genes would be regulated in a similar manner by a severe strain of CTV and CaLas, even in asymptomatic host tissues.

## Results

**Fig. 1:**
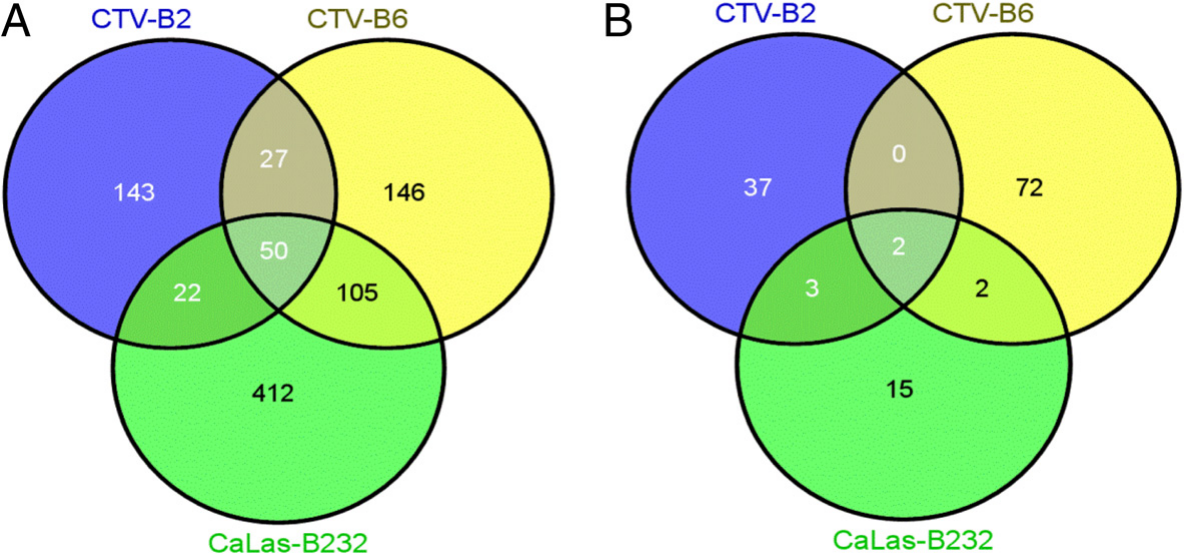
Transcripts in response to infection of *C. sinensis* with CTV-B2, CTV-B6 and CaLas-B232. Transcripts were mapped to the *C. sinensis* reference genome. **a** Number of up-regulated transcripts (**b**) Number of down-regulated transcripts

**Fig. 2:**
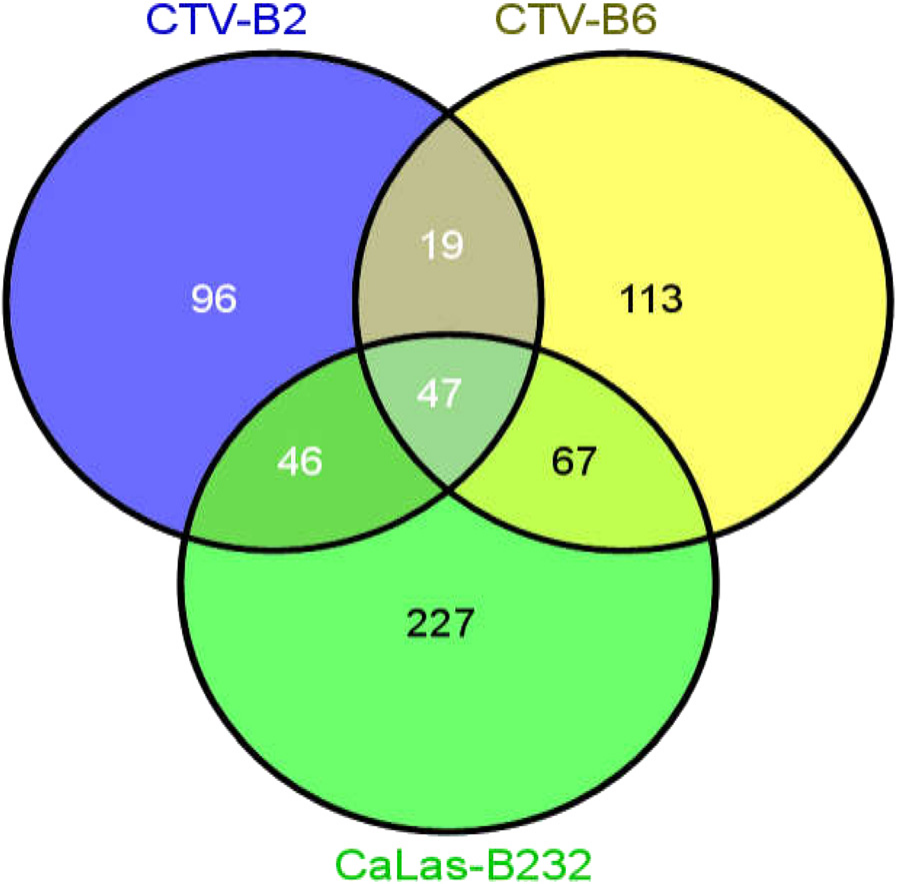
Differentially expressed genes (DEGs) in *C. sinensis* in response to infection by CTV-B2, CTV-B6 and CaLas-B232. DEGs were identified as *Arabidopsis thaliana* orthologs

Thirty-eight to 44 million clean reads were obtained from infected and control plants and mapped to the *Citrus sinensis* ‘Valencia’ reference genome [13], with approximately 73 % for CTV-B2, 76 % for the healthy, CTV-B6 and CaLa-B232 libraries (Additional file 1: Figure S1). Dramatic differences in the transcriptome profiles of sweet orange trees were observed in response to the three pathogens. Many more transcripts identified in the citrus gene database were up-regulated than down-regulated in response to the three pathogens (Additional file 1: Figure S2). More transcripts were differentially expressed in response to CaLas-B232, followed by CTV-B6 and CTV-B2 (Additional file 1: Figure S2). Among these differentially expressed transcripts (DETs), 50 transcripts were up-regulated, while only two transcripts were down-regulated in response to all three pathogens (Fig. 1). The number of transcripts regulated in response to CTV-B6 and CaLas-B232 (105 up and two down) was approximately four times the number of transcripts regulated in response to the two strains of CTV (27 up and 0 down) or CTV-B2 and CaLas-B232 (22 up and 3 down) (Fig. 1). The number of DETs responding to CaLas-B232 only (412 up and 15 down) was much larger than the number responding only to CTV-B2 (143 up and 37 down) or CTV-B6 (146 up and 72 down) (Fig. 1). A similar pattern was also found when the DETs were identified using the Arabidopsis Information Resource (TAIR) (Fig. 2). DETs with unknown functions were also identified (Additional file 1: Table S1).

**Fig. 3:**
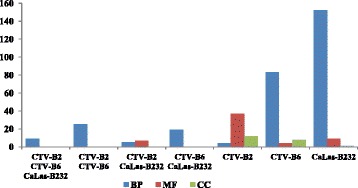
Summary of Gene Ontology categories for differentially expressed genes (DEGs). BP, biological process; MF, molecular function; CC, cellular component. The DEGs were observed in response to infection with one or more of the pathogens CTV-B2, CTV-B6 or CaLas-B232 as shown in the figure. Y-axis, number of DEGs involved in BP, MF and CC

**Fig. 4:**
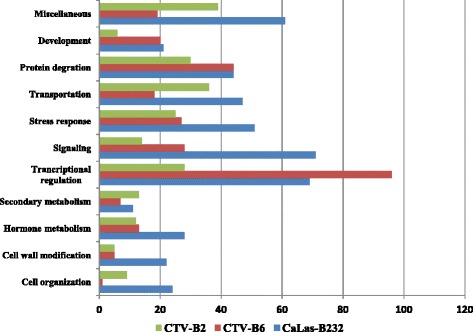
Functional categories of DEGs in response to infection by CTV-B2, CTVB6 and CaLas-B232 through Mapman. X-axis, number of differentially expressed genes involved functional categories. Y-axis, functional categories of DEGs

**Fig. 5:**
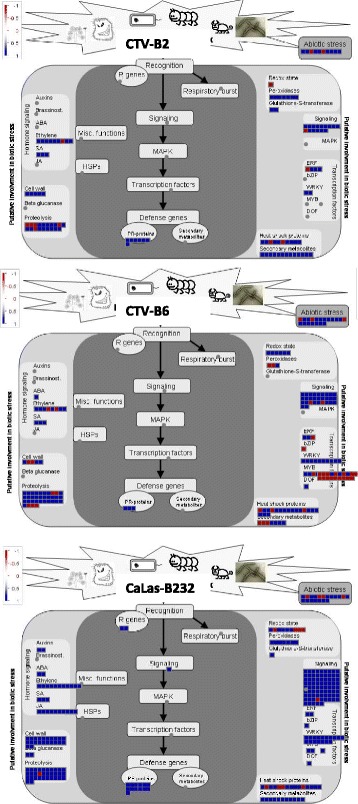
Summary of differentially expressed genes related to biotic stress in response to infection by CTV-B2, CTV-B6 and CaLas-B232

Plant Gene Sequence Enrichment Analysis (PlantGSEA) was performed with excellent *p*-values and low false discovery rates to reveal perturbed pathways. The functions of DETs observed in response to all three pathogens were mainly associated with the maintenance of circadian rhythms, such as flowering and photoperiodism, response to stimulus and response to other organism and signaling (Additional file 1: Table S2). DETs observed in response to CTV-B2 and CTV-B6 were disproportionally involved in hormone related responses, signaling pathways, and water stress (Additional file 1: Table S3). DETs observed in response to CTV-B2 and CaLas-B232 disproportionally encoded binding activities, including to iron, tetrapyrrole and heme molecules, which could affect chloroplast function, in addition to genes that respond to biotic stimulus or stress (Additional file 1: Table S4). A much larger set of DETs observed in response to both CTV-B6 and CaLas-B232 disproportionally encoded biological process (BP) functions in signaling, defense, immune and stress responses and other BPs (Additional file 1: Table S5). DETs observed to respond only to CTV-B2 were mainly associated with molecular functions (MF) and cellular components (CC) (Fig. 3) and disproportionately functioned in active transport of molecules across membranes and cell junctions (Additional file 1: Table S6). In contrast, the DETs observed to respond only to CTV-B6 or CaLas-B232 were primarily involved in BPs (Fig. 3). The long lists of DETs observed in response to CTV-B6 and CaLas-B232 are the reflection of the BPs disrupted by the severe disease symptoms caused by these pathogens, although symptoms had not yet developed in the tissues at the time they were sampled. Genes whose products functioned in transcription and inositol metabolism, as well as membrane bound intracellular organelles were up-regulated but signaling pathways were down regulated in response to CTV-B6 (Additional file 1: Table S7). The effects on inositol metabolism and membrane bound organelles could represent either the cause or an effect of chloroplast dysfunction seen in citrus infected by this pathogen, and negative regulation of signaling pathways likely impedes the defense response of the host. The long list of DETs that were observed in response only to CaLas-B232 included a large group associated with response to fungal pathogens, programmed cell death, hypersensitive responses and other host defense responses, and the production of jasmonic acid (JA) catalytic activity (Additional file 1: Table S8). The substantial number of genes annotated as ‘response to fungus’ may be correlated with the root decline and rot caused by CaLas and a potential interaction with *Phytophthora spp*. [14, 15]. Similar defense responses could also be triggered by effectors secreted by CaLas and delivered by fungus. DETs were also assigned to several specific function groups by Mapman (Fig. 4), including cell wall, hormone metabolism, secondary metabolism, transcription factors and signaling *etc*. Many pathways related to biotic stress were affected by CTV-B2, CTV-B6 and CaLas-B232 (Fig. 5). For more complete information about DETs, please refer to the supplemental information (Additional file 1: Table S9).

### RT-qPCR validation of RNA-seq results

**Fig. 6:**
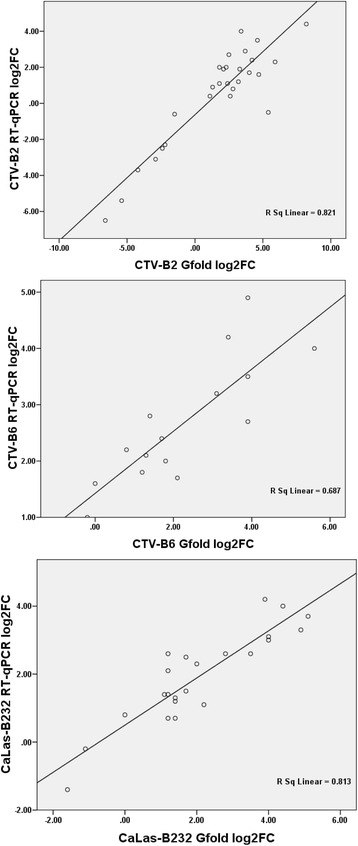
Correlation of differentially expressed genes was estimated by RT-qPCR and Gfold (log2FC). FC: Fold Change

Thirty-eight genes were tested by replicated RT-qPCR. Expression levels ranged from −7 to 8 (log_2_FC) (Additional file 1: Table S10). Spearman’s rho values, 0.821 for CTV-B2, 0.687 for CTV-B6 and 0.813 for CaLas-B232, indicated excellent correlations between Gfold analysis of the RNA Seq libraries and RT-qPCR (Fig. 6). All primers showed amplification efficiencies between 90–110 % (Additional file 1: Table S11) and melting curves showed single peaks for the evaluated genes (Additional file 1: Figure S3).

### Genes involved in cell wall and cell organization

Many transcripts related to cell wall modification and cell organization were differentially regulated, especially in response to CaLas-B232 (Additional file 1: Figure S4A). Transcripts encoding xyloglucan endotransglycosylase 6 (XTR6) were up-regulated by infection with all three pathogens (Table 1). A gene annotated as encoding a plant invertase/pectin methylesterase inhibitor (PMEI), was strongly up-regulated in response to CaLas-B232 (Table 1). Inhibition of pectin methyl esterase would strengthen the cell wall and is a common defense response [16, 17]. Another set of genes encoding expansin were up-regulated in response to CaLas-B232, in accordance with another study [10]. Expansin functions to loosen the structure of cell walls. Taken together this set of genes could contribute to the thickening and stiffening of leaves that occurs as a primary symptom of HLB and distortions of phloem cell walls as CaLas multiplies [18]. Transcripts encoding cellulose synthesis, including cellulose synthase-like B3 (CSLB3) and A2 (CSLA2), were sharply up-regulated by infection with CTV-B2 (Table 1). A transcript encoding COBRA-like protein 7 precursor (COBL7), likely involved in the deposition of cellulose, were also remarkably up-regulated by infection with CTV-B2 (Table 1), all consistent with a stronger cell wall in response to CTV-B2. A pectin lyase-like superfamily protein and beta-D-xylosidase 4 (XYL4) protein related to cell wall degradation were down-regulated by infection with CTV-B6 (Table 1). Transcripts encoding phloem protein (PP2-B13 and PP2-B15) were strongly down-regulated in response to CTV-B2 (Table 1), but up-regulated by infection with CaLas-B232. The expression of PP2-B10 gene was up-regulated by CTV-B6 and CaLas-B232 (Table 1). Because these phloem proteins move with assimilate from source to sink tissues [19], they may be markers for altered source sink flow of assimilates. Transcripts encoding NPR3 and NPR4, participating in cell organization, were also up-regulated responding to CaLas-B232 (Table 1). Transcripts encoding CYP83B1, an enzyme involved in the biosynthesis of glucosinolates and defense response by callose deposition, were up-regulated by both CTV-B6 and CaLas-B232 [20] (Table 1).

**Table 1:** DETs involved in cell wall modification and callose deposition in *Citrus sinensis* responding to CTV-B2, CTV-B6 and CaLas-B232

Gene symbol	Log2 fold change (Gfold 0.01)			*Citrus sinensis*_ID	*Arabidopsis thaliana*_ID	E-value
	CTV-B2	CTV-B6	CaLas-B232			
XTR6/XTH23	2.1	2.8	2.2	orange1.1g023204m	AT4G25810	4e-123
PMEI	0.0	0.0	3.3	orange1.1g011611m	AT5G20860	2e-110
CSLB3	3.4	0.0	0.0	orange1.1g006357m	AT2G32530	0
CSLA2	4.2	0.0	0.0	orange1.1g037406m	AT5G22740	8e-36
COBL7	3.2	0.0	0.0	orange1.1g038710m	AT4G16120	0
XYL4	−0.8	−1.2	−0.7	orange1.1g047621m	AT5G64570	0
PP2-B13	−4.2	0.0	1.4	orange1.1g042543m	AT1G56240	2e-39
PP2-B15	−6.6	0.0	1.2	orange1.1g045590m	AT1G09155	4e-57
PP2-B10	0.1	2.1	2.2	orange1.1g041394m	AT2G02360	5e-51
NPR3	0.0	0.6	1.5	orange1.1g007849m	AT5G45110	4e-175
NPR4	0.3	0.1	1.0	orange1.1g038955m	AT4G19660	2e-89
CYP83B1	0.6	2.0	1.2	orange1.1g048189m	AT4G31500	7e-79
UGT74B1	1.6	0.7	2.6	orange1.1g042896m	AT1G24100	4e-51
UGT74B1	0.0	0.9	2.9	orange1.1g036105m	AT1G24100	7e-43

*DETs* Differentially expressed transcripts

### Transcription factors (TFs)

The expression of transcription factors, including WRKYs, B-box, MYB, bHTH, heat shock transcription factors (HSF), CDR1, pseudo-response regulator (PRR), ABR1, and jumonji domain-containing protein (JMJD5), were differentially modulated in response to the three pathogens. This was especially the case in trees infected by CTV-B6 (Additional file 1: Figure S4B). Transcripts encoding PRR5 and PRR7 were up-regulated responding to all three pathogens (Table 2). A transcript encoding PRR3 was up-regulated by infection with CTV-B6 and CaLas-B232 (Table 2). Transcripts encoding JMJD5, a histone demethylase, and CCT motif family protein were up-regulated in response to both CTV strains (Table 2). JMJD5 participates in circadian systems in both plants and animals [21]. The expression level of the late elongated hypocotyl (LHY) gene was down-regulated in response to the three pathogens, especially CTV-B6 (Table 2), consistent with down-regulation in flavedo (FF) and vascular tissue (VT) of both symptomatic ‘Hamlin’ and ‘Valencia’ sweet orange infected with CaLas [22].

**Table 2:** DETs involved in transcription factors and transportation in *Citrus sinensis* infected with CTV-B2, CTV-B6 and CaLas-B232

Gene symbol	Log2 fold change (Gfold 0.01)			*Citrus sinensis*_ID	*Arabidopsis thaliana*_ID	E-value
	CTV-B2	CTV-B6	CaLas-B232			
Transcriptional factor						
PRR5	3.3	4.1	3.0	orange1.1g007462m	AT5G24470	1e-107
PPR7	1.3	1.8	1.8	orange1.1g038567m	AT5G02810	3e-29
PRR3	0.9	1.6	1.3	orange1.1g039733m	AT5G60100	8e-28
JMJD5	1.2	2.4	0.8	orange1.1g015736m	AT3G20810	4e-152
LHY1	−0.6	−1.7	−0.6	orange1.1g004250m	AT1G01060	3e-119
WRKY13	0.4	3.9	3.9	orange1.1g033112m	AT4G39410	8e-11
WRKY27	0.0	1.2	1.2	orange1.1g015616m	AT5G52830	3e-46
WRKY33	0.3	0.4	1.1	orange1.1g017783m	AT2G38470	3e-89
WRKY40	0.0	0.0	2.5	orange1.1g025097m	AT1G80840	2e-33
WRKY50	0.0	1.5	2.2	orange1.1g031482m	AT5G26170	4e-44
WRKY51	2.1	0.0	1.7	orange1.1g029257m	AT5G64810	1e-33
WRKY70	1.8	3.9	4.9	orange1.1g020291m	AT3G56400	8e-36
CDR1	0.8	3.9	5.1	orange1.1g014537m	AT5G33340	2e-119
Myb-like HTH	−5.4	0.0	1.4	orange1.1g044722m	AT5G06800	4e-31
ABR1	3.2	0.2	0.0	orange1.1g020801m	AT5G64750	3e-30
Transporter						
ZIP1	0.0	0.0	2.1	orange1.1g018182m	AT3G12750	1e-97
ZIP4	−2.2	0.0	1.2	orange1.1g014759m	AT1G10970	5e-129
ZIP5	−2.5	0.5	5.3	orange1.1g018051m	AT1G05300	1e-70
ZIP11	1.1	1.3	1.4	orange1.1g044590m	AT1G55910	4e-103
CNGC1	0.0	2.9	3.6	orange1.1g008614m	AT5G53130	3e-86
CNGC1	2.5	0.0	0.0	orange1.1g036610m	AT5G53130	1e-71
COPT1	1.2	1.1	0.3	orange1.1g042215m	AT5G59030	8e-44
COPT1	0.0	0.0	1.4	orange1.1g033054m	AT5G59030	2e-35
ABC-2	5.4	0.0	0.0	orange1.1g013741m	AT3G21090	2e-139
PHT1;7	0.1	0.3	1.8	orange1.1g010112m	AT3G54700	0
PHT1;4	1.0	1.2	0.6	orange1.1g046667m	AT2G38940	0
OPT7	0.9	2.6	1.3	orange1.1g010747m	AT4G10770	0

*DETs* Differentially expressed transcripts

Plant-specific WRKY transcription factors with a DNA-binding domain are involved in plant senescence, plant defense and response to various environmental stresses [23, 24]. Expression of WRKY13 and WRKY 70 were especially up-regulated by infection with CTV-B6 and CaLas-B232. WRKY27 and WRKY50 were also up-regulated in response to both CTV-B6 and CaLas-B232 and WRKY28, WRKY33 and WRKY40 also were up-regulated in response to CaLas-B232 (Table 2). The expression of *constitutive disease resistance* (*CDR1*) gene, encoding an aspartic protease, was very strongly induced in response to both CTV-B6 and CaLas-B232, but not at all to CTV-B2 (Table 2). *CDR1*is involved in salicylic-acid-dependent inducible resistance responses and over-expression of *CDR1* enhances resistance to bacterial pathogens in Arabidopsis [25]. A Myb-like HTH (helix-turn-helix) transcription factor was very highly down-regulated by infection with CTV-B2, but up-regulated with CaLas-B232 (Table 2). Transcripts encoding an integrase-type DNA-binding protein (ABR1), responsive to ethylene stimulus, were up-regulated by infection with CTV-B2 (Table 2).

### Transporters

Many transporter genes were differentially regulated in response to the three pathogens, especially CTV-B2 and CaLas-B232 (Additional file 1: Figure S4C). Transcripts encoding 4 zinc transporters (ZIP1, 4, 5 and 11), especially ZIP4 and ZIP5, were up-regulated in association with CaLas infection, but down regulated responding to CTV-B2, consistent with symptoms of zinc deficiency in HLB-diseased plants (Table 2). Transcripts encoding cyclic nucleotide gated channel 1 (CNGC1), involved in cyclic nucleotide or calcium transport, were differentially up-regulated in response to the three pathogens (Table 2). Transcripts encoding copper transporter 1 (COPT1) were up-regulated in response to all three pathogens (Table 2). Transcripts encoding ABC-2 type transporters were highly up-regulated by infection with CTV-B2 (Table 2). These ATP-binding cassette transporters typically function to export substrates from eukaryotic cells [26]. The expression of phosphate transporter 1;7 (PHT1;7) was induced by infection with CaLas-B232 (Table 2), consistent with a prominent role of phosphate deficiency in the HLB disease syndrome [27]. The expression of another phosphate transporter PHT1;4 was slightly up-regulated in response to both strains of CTV (Table 2). Transcripts encoding an oligopeptide transporter 7 (OPT7), involved in transport of small peptides [28], were up-regulated by infection with CTV-B6 and CaLas-B232 (Table 2).

### Hormone metabolism

Transcripts encoding jasmonic acid carboxyl methyl transferase (JMT) were highly up-regulated responding to the three pathogens (Table 3). Transcripts encoding lipoxygenase 2 (LOX2), a key enzyme in the synthesis of JA [29], and involved in the JA signaling pathway, were highly up-regulated in response to CaLas-B232, but not to the other pathogens (Table 3). Different transcripts of the senescence-related gene (SRG1), involved in ethylene metabolism, were induced in response to the three pathogens (Table 3). The expression of ethylene responsive element binding factors (ERF1 and ERF104), involved in ethylene signal transduction, was also up-regulated by infection with CaLas-B232 (Table 3). A transcript encoding a gibberellin-regulated protein was down-regulated only by infection with CTV-B6 (Table 3).

**Table 3:** DETs involved in hormone and secondary metabolism pathways in *Citrus sinensis* plants infected with CTV-B2, CTV-B6 and CaLas-B232

Gene symbol	Log2 fold change (Gfold 0.01)			*Citrus sinensis*_ID	*Arabidopsis thaliana*_ID	E-value
	CTV-B2	CTV-B6	CaLas-B232			
Hormone metabolism						
JMT	2.3	3.4	4.4	orange1.1g017514m	AT1G19640	4e-91
LOX2	0.6	0.7	4.0	orange1.1g002670m	AT3G45140	0
SRG1	1.6	0.0	0.0	orange1.1g040640m	AT1G17020	8e-60
SRG1	0.6	1.2	0.8	orange1.1g035879m	AT1G17020	9e-113
SRG1	0.6	0.3	1.1	orange1.1g045260m	AT1G17020	1e-119
ERF-1	0.0	1.6	2.1	orange1.1g028566m	AT4G17500	7e-36
ERF104	0.0	0.3	1.9	orange1.1g023209m	AT5G61600	4e-41
^a^(Gibberellin)	0.0	−1.6	−0.6	orange1.1g035431m	AT5G14920	3e-22
Secondary metabolism						
2OG-Fe(II)	4.2	4.1	7.0	orange1.1g019027m	AT4G10490	4e-141
DMR6	1.9	2.8	3.5	orange1.1g019665m	AT5G24530	3e-139
2OG-Fe(II)	8.9	0.0	0.0	orange1.1g039524m	AT3G13610	1e-126
TPS21	8.2	0.0	0.0	orange1.1g041926m	AT5G23960	8e-111
TPS21	0.0	−1.1	0.0	orange1.1g010399m	AT5G23960	7e-75
TPS21	0.0	0.0	2.2	orange1.1g043754m	AT5G23960	2e-68
OMT1	2.1	0.0	0.1	orange1.1g017595m	AT5G54160	7e-93
OMT1	0.1	1.3	0.4	orange1.1g044458m	AT5G54160	6e-99
GGPS1	3.9	0.0	1.0	orange1.1g020845m	AT4G36810	5e-19
VTE2	2.7	0.0	0.0	orange1.1g029089m	AT2G18950	2e-41
VTE2	0.0	−1.5	−0.5	orange1.1g013845m	AT2G18950	2e-66
^a^(Terpenoidcycl)	7.2	0.0	0.0	orange1.1g044462m	AT3G25810	1e-97
LAC4	0.0	0.5	3.1	orange1.1g008799m	AT2G38080	0
LAC5	0.0	0.7	2.7	orange1.1g008066m	AT2G40370	0
LAC7	0.0	0.0	2.2	orange1.1g008366m	AT3G09220	0
UGT74B1	1.6	0.7	2.6	orange1.1g042896m	AT1G24100	4e-51

^a^Genes without abbreviations

*DETs* Differentially expressed transcripts

### Secondary metabolism

Many different transcripts encoding 2-oxoglutarate and Fe (II)-dependent (2OG-Fe (II)) oxygenases were highly up-regulated in response to all three pathogens (Table 3). Transcripts encoding terpene synthase (TPS21), involved in isoprenoid and terpenoid metabolism, were induced up to eight fold by infection with CTV-B2, and to a lesser extent infection by CaLas-B232, but were repressed by CTV-B6 (Table 3). These perturbations of the terpenoid pathways likely contribute to the different volatile components detected following infection by CTV or CaLas [30, 31]. The expression of O-methyltransferase (OMT), participating in flavonoid and phenylpropanoid metabolism, was up-regulated in response to both strains of CTV (Table 3). A transcript encoding geranylgeranyl pyrophosphatesynthase1 (GGPS1), also involved in isoprenoid biosynthetic process, was strongly up-regulated in response to CTV-B2 and less so to CaLas-B232 (Table 3). Transcripts encoding homogentisate phytyltransferase (VTE2) were up-regulated by infection with CTV-B2, but were down-regulated by infection with CTV-B6 and did not respond to CaLas-B232 (Table 3). VTE2 catalyzes the synthesis of tocopherol, which plays an important role in protecting chloroplast membranes. The expression of VTE2 was induced in symptomatic and repressed in asymptomatic leaves of sweet orange in response to CaLam [32]. Transcripts encoding terpenoidcyclases/proteinprenyl transferase were also significantly up-regulated by infection with CTV-B2 (Table 3). Laccase genes *LAC4*, *LAC5* and *LAC7* participating in simple phenol metabolism, were over-expressed in response to CaLas-B232 (Table 3). Transcripts encoding UDP-glucosyl transferase 74B1 (UGT74B1) involved in plant secondary metabolism and playing a potential role in stress or defense responses were up-regulated by both CTV-B2 and CaLas-B232 [33] (Table 3).

### Signaling

Plant receptor-like proteins/kinases (RLPs/RLKs) regulate developmental processes and respond to pathogens and abiotic stresses [34]. A large number of transcripts encoding RLRs/RLKs, including leucine rich repeat (LRR) kinases, wall-associated kinases (WAKs) and Domain of Unknown Function 26 (DUF26) kinases, were differentially expressed in response to all three pathogens, and very notably to CaLas-B232 (Additional file 1: Figure S4F). The expression of LRR kinases were up-regulated responding to CTV-B6 and CaLas-B232 but not to CTV-B2, consistent with their role in defense response [34]. These included RLP6, RLP9, RLP13, RLP14, RLP15 and RLP45, which were consistently up-regulated by infection with CaLas-B232 and CTV-B6 (Table 4). Transcripts related to wall associated kinases were differentially expressed in response to CTV-B2 and CaLas-B232, including WAK2 and WAK7 (Table 4). Notably, the expression of two members of DUF26 RLKs, cysteine-rich RLK2 (CRK2) and receptor-like kinase1 (RLK1), was significantly induced by infection with CTV-B2 (Table 4). The expression of E1-E2 ATPase was up-regulated in response to all three pathogens. The expression of EF-hand binding protein and calmodulin (CaM) was induced responding to CTV-B6 and CaLas-B232 (Table 4). Transcripts encoding cold-circadian rhythm RNA binding-like protein (CCR-like, CCL) were up-regulated in response to all three pathogens, especially CTV-B6, approximately five fold (Table 4).

**Table 4:** DETs involved in signaling, stress response and miscellaneous pathways in *Citrus sinensis* plants infected with CTV-B2, CTV-B6 and CaLas-B232

Gene symbol	Log2 fold change (Gfold 0.01)			*Citrus sinensis*_ID	*Arabidopsis thaliana*_ID	E-value
	CTV-B2	CTV-B6	CaLas-B232			
Signaling						
RLP6	0.0	1.3	1.8	orange1.1g046844m	AT1G45616	1e-115
RLP9	0.0	1.4	2.7	orange1.1g021196m	AT1G58190	8e-23
RLP13	0.0	3.1	3.6	orange1.1g044924m	AT1G74170	2e-92
RLP14	0.0	2.2	3.0	orange1.1g028394m	AT1G74180	5e-16
RLP15	0.0	2.4	3.4	orange1.1g040699m	AT1G74190	1e-73
RLP45	0.3	2.2	2.5	orange1.1g040468m	AT3G53240	2e-85
WAK2	−1.4	−0.5	−1.1	orange1.1g004702m	AT1G21270	7e-167
WAK2	0.0	0.2	1.0	orange1.1g036131m	AT1G21270	7e-102
WAKL7	0.0	0.0	2.1	orange1.1g023486m	AT1G16090	1e-17
CRK2	4.0	0.0	0.0	orange1.1g006168m	AT1G70520	5e-102
RLK1	3.3	0.0	0.0	orange1.1g047157m	AT5G60900	4e-106
ATPase E1-E2	1.6	2.5	2.8	orange1.1g047874m	AT3G63380	0
Ca EF-hand	0.0	2.2	4.1	orange1.1g027442m	AT4G13440	1e-21
Calmodulin	0.0	1.8	3.1	orange1.1g011961m	AT1G73805	1e-125
CCL	2.2	5.0	1.0	orange1.1g033578m	AT3G26740	6e-20
Stress response						
HSP70	0.2	2.1	2.3	orange1.1g005824m	AT5G02500	1e-175
HSP81-1	-0.9	−1.2	−1.1	orange1.1g005370m	AT5G52640	0
Kunitz	0.0	0.0	5.4	orange1.1g028238m	AT1G17860	4e-28
ACD2	4.7	0.0	0.0	orange1.1g029930m	AT4G37000	3e-39
PA2	2.2	0.0	0.0	orange1.1g018873m	AT5G06720	5e-95
WCRKC1	0.0	3.4	0.0	orange1.1g030114m	AT5G06690	2e-49
^a^(Oxidoreductase)	0.0	2.0	1.5	orange1.1g025278m	AT1G23740	3e-84
Miscellaneous						
GI,FB	3.7	5.6	3.5	orange1.1g001216m	AT1G22770	0
NAC2	2.0	3.9	4.5	orange1.1g013077m	AT5G04410	4e-36
UDP-Glycosyl	2.9	0.0	0.0	orange1.1g043304m	AT5G12890	2e-109
UDP-Glycosyl	0.4	0.0	1.7	orange1.1g012474m	AT3G11340	2e-133
CYP706A4	2.5	3.5	2.9	orange1.1g009495m	AT4G12300	5e-114
ConA	5.9	0.0	0.6	orange1.1g024824m	AT4G04960	1e-57

^a^Genes without abbreviations

*DETs* Differentially expressed transcripts

### Stress response

Many disease resistance (R) genes were differentially regulated in response to CTV-B2 and CaLas-B232, and fewer to CTV-B6 (Additional file 1: Figure S4G), including CC (TIR)-NB-LRR (coiled-coil/Toll-interleukin1 receptor-nucleotide binding-Leucine rich repeat) and NB-ARC (nucleotide binding-adaptor shared by APAF-1, certain *R* gene products and CED4) domain-containing disease resistance proteins, the largest and well-characterized group of R genes. Transcripts encoding heat shock proteins (HSPs) were especially regulated in response to CTV-B6 and CaLas-B232 (Table 4). The expression of Kunitz-type protease inhibitor (PI), located in the cell wall and responsive to peroxidase, was significantly up-regulated only in response to CaLase-B232 (Table 4). Remarkably, a transcript encoding accelerated cell death 2 (ACD2) was highly up-regulated only responding to CTV-B2 (Table 4).

### Oxidation/reduction processes

Transcripts related to the peroxidase super family were also differentially regulated in response to the three pathogens (Additional file 1: Table S9). Peroxidase 2 (PA2) mRNA accumulated in response to CTV-B2 (Table 4). Transcripts encoding WCRKC thioredoxin1 (WCRKC1), likely involved with redox homeostasis in the chloroplast, were greatly up-regulated only responding to CTV-B6 (Table 4). Transcripts encoding oxidoreductase, a zinc-binding dehydrogenase family protein, were up-regulated in response to CTV-B6 and CaLas-B232 (Table 4).

### Development, protein degradation and miscellaneous functions

Transcripts encoding thiazole biosynthetic enzyme (THI1, TZ, THI4) were up-regulated responding to all three pathogens, especially CTV-B6 (Additional file 1: Table S9). Thiamine biosynthetic genes respond to bacteria, and also act in starch and maltose metabolism [35]. These enzymes may contribute to the accumulation of starch in leaves of diseased trees. Transcripts encoding gigantea (GI) and NAC domain containing protein 2 involved in the development and strengthening of secondary cell walls were strongly up-regulated in response to all three pathogens (Table 4). A set of transcripts involved in protein degradation were also differentially regulated in response to the three pathogens (Additional file 1: Table S9), consistent with a previous result that protein degradation and misfolding processes were activated in citrus fruits infected with CaLas [36]. A larger set of transcripts encoding UDP-glycoyl transferase and cytochrome P450 were differentially regulated in response to all three pathogens (Additional file 1: Table S9). Both UDP-glycoyl transferase and cytochrome P450 are involved in secondary metabolism, responses to biotic and abiotic stress and oxidation/reduction process. A transcript encoding a concanavalin A-like (ConA-like) protein with a jacalin-like lectin domain was very highly up-regulated by infection with CTV-B2 (Table 4). Jacalin-related proteins were demonstrated as components in plant defense system [37].

## Discussion

Recently, global analysis of gene expression has been applied widely to study plant-pathogen interactions. Though studies have been carried out to identify DETs in citrus infected with CaLas and CTV by microarray or RNA-seq, these studies have been conducted with each pathogen separately or in the context of susceptible *vs*. tolerant plant genotypes [9, 10, 12, 38, 39]. In the present study, RNA-seq was used to identify DETs in susceptible ‘Valencia’ sweet orange after CaLas and CTV infection, with all plants grown and inoculated together in the same greenhouse. We found many biological processes and pathways affected by the three pathogens. The number of DETs reflected the complexity of symptoms in response to the three pathogens. The transcriptome profiles were more similar between CTV-B6 (VT genotype) and CaLas-B232 than between the two CTV genotypes CTV-B2 (T30 genotype) and CTV-B6 or between CTV-B2 and CaLas-B232. This is consistent with similar symptoms. CTV-B6 causes severe stem pitting [2, 3], which is a major disturbance in the phloem system and CaLas-B232 induces typical symptoms of HLB which include degeneration of phloem cells. Both disease syndromes also include yellowing and cupping of leaves caused by the failure of phloem transport and starch accumulation in the leaves. These symptoms are not induced by CTV-B2, although the pathogen is fully systemic in the host.

### Circadian system affected by all three pathogens

**Fig. 7:**
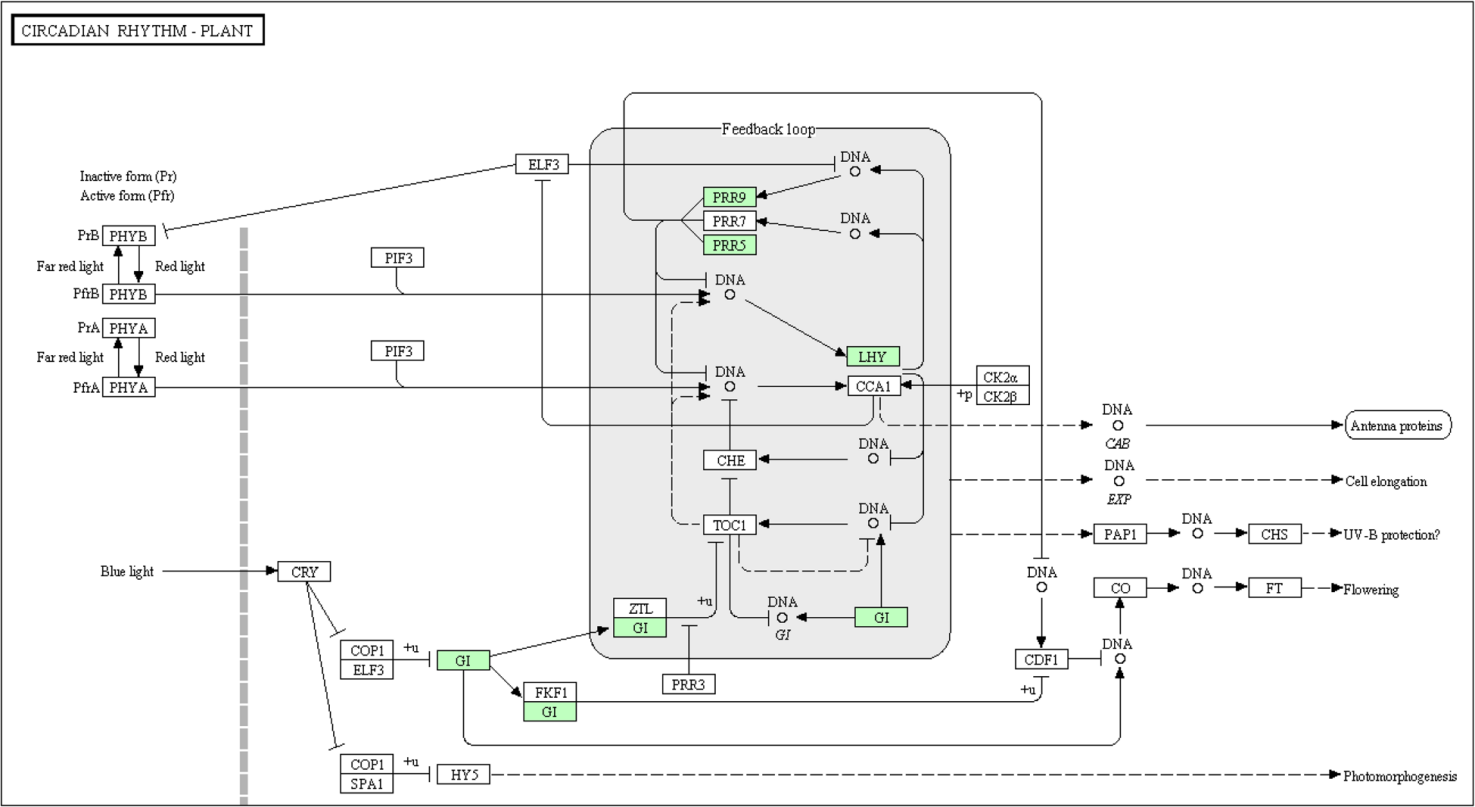
Circadian rhythm pathway regulated by CTV-B2, CTV-B6 and CaLas-B232. The green boxes represent genes that were differentially expressed in response to all of the three pathogens

The circadian system, an endogenous mechanism vital to organisms, allows them to coordinate physiological changes to predictable day/night cycles and plays extremely important roles in reproductive development, germination, growth and carbohydrate metabolism processes throughout the life cycle of the plant [40, 41]. The circadian rhythm regulates the expression of 31 % of the *Arabidopsis* genome and have been found to be significantly disrupted by abiotic and biotic stress [42]. The circadian clock controls the cotton plant’s (*Gossypium hirsutum*) sensitivity to cold [43] and also regulates the transcription levels of some pathogen response related genes in Arabidopsis [44]. Disruption of the circadian rhythms by CTV or CaLas has not been reported previously, but off season flowering is observed in citrus groves in response to CTV and HLB [45–47]. In our study, the circadian system was heavily affected by the three pathogens (Fig. 7), especially CTV-B6. Circadian clock-associated components PRR5 and PRR7 have been proven to play important roles in the circadian rhythm by repressing the expression of *CCA1* (circadian clock-associated 1) and *LHY* (late elongated hypocoty) [48], consistent with the up-regulation of *PRR* and down-regulation of *LHY* genes in our study. CCA1 and LHY are homologous to Myb-related DNA-binding proteins and the circadian clock components form an auto-regulatory feedback loop at the transcription levels [49, 50] (Fig. 7). Accumulation of PRR1/TOC1 (timing of cab expression 1) in late day and early night promotes the rise of CCA1 and LHY proteins during the early and mid day, which in turn represses transcription of *PRR1/TOC1*. TOC1 also acts in concert with JMJD5 to regulate the circadian clock [21]. PRR family proteins and C-terminal (CCT) motif proteins are homologous and contribute to the control of the circadian rhythm that controls photoperiodic flowering [51]. The circadian clock controls the stability of the CCL mRNA at different times of day [52] as well as the expression of the GIGANTEA (*GI*) gene. The CCL gene was up-regulated in juice vesicles (JV) of ‘Hamlin’ and ‘Valencia’ by infection with CaLas [22], consistent with our study. The *GI* gene encodes a protein with six putative membrane-spanning domains that regulates both photoperiodic flowering and the deposition of wall ingrowths in phloem parenchyma transfer cells in *A. thaliana* [11, 18, 53, 54]. The formation of wall ingrowths in transfer cells plays an important role in phloem biology by producing physical barriers to limit pathogen invasion [55]. GI is also necessary to maintain the appropriate period of *CCA1* and *LHY* gene expression and the circadian amplitude in the outer feedback loop in *A. thaliana* [56]. Therefore, the change of gene expression induced by all three pathogens, but especially by CTV-B6, indicated the alteration of the circadian cycle in the affected plants. Because of the large number of genes coordinated by the circadian clock [42], plants infected with these pathogens may have other abnormal gene expression patterns.

### Cell wall modification defense response

Plant cell walls are composed of cellulose, hemicelluose and pectins and play a vital defensive role in plants against pathogens. Pectin is found in primary cell walls and the middle lamella, where it contributes to cell wall structure and binds cells together [57, 58]. Extensive disassembly of pectin is associated with tissue deterioration, leaf abscission [59], as well as swelling of the middle lamella [60], which was observed in cell walls surrounding sieve elements (SEs) at early stages of infection with CaLas [18]. Pectin hydrolysis often occurs in response to soft rot bacteria and fungi and pectin methylesterase inhibitors (PMEI) play a role in defense by regulation of cell wall susceptibility to microbial endopolygalacturonases. The expression of PMEI was also up-regulated in ‘Valencia’ leaves in association with CaLas [10], as well as in present study. The over expression of PMEI may also be associated with the interaction with *Phytophthora spp*. [14, 15] or opportunistic fungi.

Xyloglucan endotransglycosylase (XET) is an enzyme involved in the synthesis of xyloglucan, the predominant hemicellulose in the cell walls. Transcripts encoding XET were up-regulated in *C. sinensis* leaves [10] but down-regulated in *C. sinensis* stems infected with CaLas [39]. XET plays a profound role during the process of fruit ripening [61], which is impaired by CaLas. *A. thaliana XTH33*, encoding a xyloglucan endotransglycosylase/hydrolase (XTH), protects plants against aphids by modifying the cell wall [62]. Tomato *leXTH1* mRNA accumulated in response to dodder, *Cuscuta reflexa* [63], and the expression of *Medicago truncatula MtXTH1* gene was induced in association with *Glomus* and *Gigaspora* in roots [64]. Genes related to cellulose synthesis, *CSLC7* and *CSLD4*, were down-regulated in stems in association with CaLas [39]. In the current study, the expression of cellulose synthase-like genes *CSLB3*, *CSLA2* and *COBL7,* was strongly up-regulated by infection with CTV-B2, but not by infection with CaLas and CTV-B6. CSLA proteins have mannan/glucomannan synthase activity and mannans have vital roles in metabolic networks that regulate cell wall structure, carbohydrate storage and other cellular processes in plants [65, 66]. *AtCSLA7* is essential for synthesis of a cell wall β-linked polysaccharide throughout the plant [67]. Both *CSLH* from barley and *CSLF* from rice mediate the synthesis of cell wall (1,3 and 1,4)-β-D-glucans in transgenic *A. thaliana* [68, 69]. COBRA-like (COBL) genes, predicted to encode a putative glycosylphophatidylinositol (GPI)-anchored protein in *A. thaliana* [70], play an important role in cell wall maintenance and biosynthesis, controlling the orientation of cell expansion and modulation of cellulose deposition in the cell walls of roots [71, 72]. Another COBRA-like protein, Brittle culm1 (BC1), provides mechanical strength for rice by regulating the biosynthesis of secondary cell walls [73]. Therefore, the plant responded to CTV-B2, but not to CTV-B6 and CaLas by activating enzymes that would work together to strengthen the cell wall, which may contribute to the reinforcement of physical barriers to restrict the invasion of CTV-B2.

### Phloem damage and defense response

PP2, one of the well-known sieve element (SE) proteins, was found in plugged sieve pores and was described in cucurbit phloem sap [74]. Both CTV-B6 and CaLas-B232 damage phloem tissue and similar sets of genes may be altered by both pathogens. Several studies have reported that transcripts encoding phloem proteins (PP2-B10, PP2-B14 and PP2-B15) were up-regulated in symptomatic leaves in response to CaLas [11, 22, 32]. PP2 accumulated at the sieve plate linked to symptom development in response to CaLas [11]. Cucumber csPP2 interacts with the *Hop stunt viroid* (HSVd) RNA and may facilitate its long-distance movement [75, 76]. PP2 also can interact with polyadenylated mRNAs [75]. The altered expression of PP2 genes was tested and confirmed by RT-qPCR (Additional file 1: Table S10). The closure of sieve pores by PP2 is normally accompanied by the accumulation of callose on sieve plates under abiotic or biotic stress [77].

Callose is widely distributed in higher plants and is deposited at cell plates during cytokinesis [78] and in plasmodesmata, and contributes to the regulation of movement of molecules from cell-to-cell [79]. PP2 and callose were deposited at the sieve plates in apple trees infected with the phloem-limited pathogen ‘*Ca.* Phytoplasma mali’ [80]. Accumulation of PP2 and callose proteins may provide a physical barrier against further invasion of CTV-B6 and CaLas-B232, and contribute to long-distance signaling [10, 19, 75]. The over expression of callose and PP2 genes is consistent with the phloem damage and defense mechanisms activated by CTV-B6 and CaLas-B232. The clear down regulation of PP2 by infection with CTV-B2 suggests that CTV-B2 was not perceived by the host plant in a way that triggered the defense response.

### Ions and nutrient deficiency

The expression of both EF-hand calcium-binding and calmodulin-binding proteins was down-regulated in stems of *C. sinensis* inoculated with CaLas [39], while the expression of EF-hand calcium-binding protein was up-regulated about 5 fold in *C. sinensis* 13–17 weeks after inoculation with CaLas [10]. In the current study, the expression of both genes was also up-regulated (Table 4). The differences among these studies might be attributed to the different stages of infection or tissues used. CNGC (cyclic nucleotide gated channel) mRNA accumulated in flavedo (FF), vascular tissue (VT) and juice vesicles (JV) of symptomatic ‘Hamlin’ and ‘Valencia’ sweet orange infected with CaLas [22]. Our transcriptome profiles showed up-regulation of EF-hand calcium-binding protein, calmodulin-binding protein, CNGCs and ATPase E1-E2 proteins in young leaves in response to CaLas. ATPase E1-E2 accumulated in response to CTV-B2 and EF-hand calcium-binding proteins accumulated in response to CTV-B6. ATPase E1-E2 is involved in Ca^2+^-translocation and CNGCs are involved in Ca^2+^-signaling processes and both maintain [Ca^2+^] homeostasis in the plant [81, 82]. In Arabidopsis, CNGC1 conducts Ca^2+^ into cells [83] and AtCNGC2 responds to pathogen recognition by increasing [Ca^2+^]_cyt_ levels [84]. AtCNGC4 is also an essential component of the signaling pathway leading to the hypersensitive response [85]. Calmodulin (CaM) is a major Ca^2+^ signal transducer in both animals and plants [86] and is involved in Ca^2+^-mediated plant disease resistance responses [87]. Soybean CaM genes *SCaM4* and *ScaM5* were induced within 30 min by pathogens [87]. Evidence of altered calcium metabolism is also demonstrated by the formation of crystal idioblasts in CaLas infected plant tissues [18]. These idioblasts are believed to contain calcium oxalate (CaOX), which can serve dual functions in the regulation of calcium concentration and plant defense. An influx of Ca^2+^ can also activate Ca^2+^-dependent defense responses such as PP2 aggregation and callose deposition [80]. The accumulation of EF-hand calcium-binding protein, CaM, ATPase E1-E2 and CNGC and the formation of CaOX crystals suggest imbalances in calcium metabolism after infection by the three pathogens. Ca^2+^-dependent defense mechanisms were activated by the three pathogens and play more significant roles in defense response to CaLas-B232.

Zinc transporters such as ZIP1, ZIP2, ZIP3, ZIP4, ZIP5 and ZIP11, are induced in plants in response to zinc deficiency. ZIP4 and ZIP5 mediate zinc uptake from the rhizosphere and are responsible for translocation of zinc within the plant [88, 89]. Citrus plants affected by CaLas present nutritional deficiency symptoms typically associated with zinc deficiency [4, 5]. Up-regulation of ZIP1 was observed in transcriptome profiles of ‘Madame Vinous’ and ‘Navel’ sweet oranges inoculated with CaLas [9], and over expression of ZIP5 was also found in the susceptible genotype ‘Cleopatra mandarin’ but not in the tolerant genotype ‘US-897’ inoculated with CaLas [38]. In another study, transcripts encoding ZIP1, ZIP4 and ZIP5 were up-regulated in ‘Pera’ sweet orange inoculated with CaLam [32], all of which is consistent with the over expression of four zinc transporters (ZIP1, ZIP4, ZIP5 and ZIP11) in our study. The up-regulation of zinc transporters in susceptible citrus genotypes infected with CaLas or CaLam in the late stages is consistent with the zinc deficiency symptoms seen on leaves, but is also observed in young leaves without symptoms. If CaLas-infected plants experience dramatic fibrous root decline before foliar symptoms appear [14, 15], this would limit the uptake of sufficient microelements such as Zn and Cu from the rhizosphere. CaLas also assimilates zinc from the plant phloem sap with assistance of zinc uptake ABC transporters [90], further contributing to the zinc deficit in the host.

Zinc deficiency leads to a wide range of ultrastructural problems with chloroplasts, mitochondria, cytoplasm and aberrant regulation of DNA-transcription and RNA-processing in plants [91], followed by foliar symptoms on leaves and delayed maturation of fruits. Transcription of Zn and Cu transporters would then be up-regulated in response to CaLas to meet the deficits of Zn, Cu and other micronutrients. This also may explain why HLB disease severity was reduced by the application of various nutritional products including micronutrients on the foliage extended the vigor and productivity of CaLas infected trees [6]. CaLas encodes a zinc-dependent metalloprotease (Accession Number: ACT57246; GI: 204040450) similar to RseP-like site-2 proteases (S2P), which need zinc to enhance stability [92]. The introduction of zinc-dependent metalloproteases into organisms can cause impairment of physiological functions that lead to disease or death [93]. The zinc-dependent metalloprotease in CaLas may explain the up-regulation level of zinc transporters to a greater degree than transporters of other microelements in response to CaLas, and also the higher expression level of zinc transporters than in plants infected with CTV-B6. In contrast, ZIP4 and ZIP5 were notably down-regulated in asymptomatic sweet orange trees infected by CTV-B2 in this study. Taken together, the differential expression of Zn, Cu and Ca transporters is evidence of impaired homeostasis in plants caused by the three pathogens. The greater expression level of zinc transporters in response to CaLas than CTV-B6 may be attributed to root decline and usage of zinc by CaLas.

### Hormone-mediated defense response

The JMT gene was induced in young leaves and in mature fruits infected with CaLas [94], consistent with up-regulation in our study (Table 3). JMT catalyzes the synthesis of methyl jasmonate (MeJA), which plays an important role in JA-regulated plant defense responses [95]. In our study, the up-regulation of *LOX2* encoding a lipoxygenase involved in the JA pathway responding to CaLas is in accordance with previous study [10]. The transcription level of *LOX* was also increased in *Citrus reticulata* inoculated with *Xylella fastidiosa* at the early stage [96]. Many WRKY transcription factors are involved in hormone-mediated signaling pathways under biotic and abiotic stress [97]. WRKY70 coordinates the cross talk of SA-and JA-mediated signals in plant defense, and over expression of WRKY70 increased resistance to pathogens and influenced expression of SA induced pathogenesis-related genes [98]. WRKY6, WRKY40 and WRKY70 were up-regulated about five fold in symptomatic leaves of sweet orange [32] and WRKY70 was expressed at a higher level in fruits than in leaves in response to CaLas [94]. The accumulation of WRKY13 transcripts affected transcription of more than 500 genes, some that functioned in disease resistance [99]. The expression of WRKYs may also link to extensive transcriptional repression and activation by its own WRKY members [97]. Hormone-mediated defense responses were activated by all three pathogens. However, hormone-mediated defense responses were stronger in response to CTV-B6 and CaLas-B232 than the mild strain CTV-B2 which did not trigger these defense responses to nearly the same degree.

### Secondary metabolism related defense

Plant secondary metabolites play an important role in defense responses to herbivores, pests and pathogens [100]. 2OG-Fe (II) oxygenases, involved in the production of plant secondary metabolites such as flavonoids, were induced by the three pathogens. DMR6, encoding a 2OG-Fe (II) oxygenase, plays a role in plant defense in *A. thaliana* [101]. The expression of 2OG-Fe (II) oxygenase was up-regulated in the susceptible genotypes (*C. sinensis* and *C. reticulata*), and also extremely up-regulated in the tolerant genotype (US-879, *C. reticulata x P. trifoliata*) infected with CaLas [10, 38]. Terpene synthases and terpenoid cyclases were highly induced in response to CTV-B2 and both are involved in the production of terpenes, the largest class of secondary metabolites [102], which are important factors in resistance to several pathogens and pests [103]. Hence, the expression of secondary metabolites was triggered by all three pathogens, but to a much greater degree in response to CTV-B2.

### Pathogenesis related defense responses

Receptor-like proteins/kinases (RLPs/RLKs) are important factors in hormone signaling in response to abiotic and biotic stress in plants. More RLPs/RLKs were induced in response to CTV-B6 and CaLas-B232 than to CTV-B2. However some RLPs/RLKs, such as CRK2 and RLK1, were only induced in response to CTV-B2. These are cysteine-rich RLKs (CRKs) of the DUF26 group. Three CRKs (CRK4, CRK19 and CRK20), closely related to CRK5, were also induced by SA treatment and infection with *Pseudomonas syringae* and all activate rapid cell death in transgenic Arabidopsis [104]. CRK genes, *PRK1* to *PRK4* from Potato and *PK20-1* from common bean were differentially regulated in response to symbionts, elicitor molecules and pathogens [105]. More pathogenesis-related (PR) genes were up-regulated in sweet orange inoculated with CaLas-B232 than with CTV-B2 and CTV-B6.

Transcripts encoding Kunitz-type trypsin inhibitors were up-regulated in response to CaLas. The Kunitz trypsin inhibitors are responsive to wounding [106] and protease inhibitors from plants may also inhibit the growth of a variety of pathogenic bacteria and fungi. An important gene to note is *ACD2,* up-regulated only in response to CTV-B2. The product of *ACD2* has extensive similarities to red chlorophyll catabolite reductase, which is involved in the degradation of the porphyrin component of chlorophyll (Chl) [107]. Over expression of *ACD2* in plants induced many characteristics of the hypersensitive response (HR), including cell wall modification, accumulation of mRNA of PR genes and resistance to bacteria [108]. The up-regulation of *ACD2* did not restrict the growth of *Pseudomonas syringae* but reduced disease symptoms [108, 109], which may explain symptom suppression in response to CTV-B2. Pathogenesis-related defense responses were induced by all three pathogens, and plants used more kinase signaling and stress response pathways in response to CaLas-B232 than to either strain of CTV.

## Conclusions

More genes and pathways were regulated in response to CaLas-B232, and to a lesser extent CTV-B6, than to CTV-B2, consistent with the symptoms caused by corresponding pathogens. Gene expression patterns of trees infected with CTV-B6 and CaLasB232 were more similar than those of infected with CTV-B2 and CTV-B6. These gene expression patterns in the asymptomatic leaves sampled lead to similar physiological changes, such as phloem damage, root decline, yellowing and cupping of leaves caused by both CTV-B6 and CaLas-B232. The circadian rhythm system of sweet orange plants was heavily perturbed by all three pathogens, most notably by CTV-B6, and ion balances were also disrupted by all three pathogens, but especially by CaLas-B232. Many defense responses were triggered by all three pathogens but with different preferences: Defense responses based on cell wall modification and secondary metabolism were activated by all three pathogens, but were more prominent in response to CTV-B2. Defense responses modulated by transcription factors responded most significantly to CTV-B6. Ca^2+^-dependent, kinase signaling and pathogenesis-related defense responses functioned more actively in response to CaLas-B232 than to either strain of CTV. DETs regulated by CTV-B2, but not by CTV-B6 or CaLas, are likely to be important to protect the plant from CTV-B2, based on perception of the pathogen by the host and efficient triggering of systemic defense responses.

## Methods

### Inoculation of experimental trees with CTV and CaLas

CTV mild strain B2 (T30 genotype, Florida), severe strain B6 (VT genotype, =SY568, California) and CaLas strain B232 (Thailand) are maintained *in planta* as part of the Exotic Pathogens of Citrus Collection (EPCC) at the USDA-ARS Beltsville Agricultural Research Center (BARC) in Beltsville, MD. Experimental seedling trees were ‘Valencia’ sweet orange. Each pathogen was inoculated into three replicate trees by inverted T-bud grafting when the trees were actively growing. Three trees were also mock-inoculated with healthy buds as the control. The trees were maintained in a greenhouse with supplemental fertilization provided with the irrigation water as described previously [110].

### Detection of CTV and CaLas with PCR

In order to confirm that the plants were successfully inoculated and infected with the phloem-limited pathogens, RNA and DNA were extracted from leaf midribs. DNA was extracted from 0.1 g of leaf midrib using the Plant DNeasy® Mini Kit (Qiagen, Valencia, CA) according to the manufacturer’s instructions. qPCR assays were performed as described [111] with a SmartCycler (Cepheid, Sunnyvale, CA). Total RNA was extracted with Trizol (Invitrogen, Carlsbad, CA) and treated with TURBO™ DNase (AM2239, Ambion) according to the manufacturer’s instructions. cDNA synthesis was done with the Thermoscript RT-PCR System for First-Strand cDNA Synthesis (Invitrogen). CTV cDNA (2 μl as template) was amplified in a 25 μl reaction volume containing 10 X reaction buffer, 10 mM dNTP, 25 mM MgCl_2_, 10 μM of each primer [112] and 0.3 U of *Taq* polymerase. Amplification was carried out with a PTC-200 thermal cycler (MJ Research) as follows: 94 °C for 3 min, then 94 °C for 30 s, 57 °C for 40 s, and 72 °C for 40 s. After 30 cycles the reactions were incubated an additional 10 min at 72 °C prior to storage at 4 °C. Products were analyzed by electrophoresis on 1.5 % agarose gels and stained with Gel Red (Biotium, Hayward, CA). Plants with confirmed infections were used for transcription analysis.

### Isolation and quantification of total RNA

After infection by the pathogens had been confirmed by PCR and qPCR, but before symptoms became evident, RNA samples were collected in the morning (8–10 AM) from young, soft, not fully expanded leaves of uniform size at 32 weeks after inoculation (Additional file 1: Figure S5). Total RNA was extracted and treated as described above. A portion of the RNA samples from three PCR-positive independent biological trees of each pathogen and from mock-inoculated trees were pooled. The quantity and quality of total RNA was determined with the Qubit 2.0 Fluorometer (Invitrogen) and the Bioanalyzer 2100 (Agilent Technologies, Santa Clara, CA), respectively. A total of 30 μg of RNA (RIN ≥ 7.5) [113] from healthy ‘Valencia’ sweet orange and ‘Valencia’ sweet orange infected with CTV-B2, CTV-B6 and CaLas-B232 were sent to SeqWright, Inc. (Houston, TX, USA) for paired-end sequencing of polyA selected mRNA with the Illumina HiSeq 2500 platform. The remainder of the RNA extracts was retained as replicates for RT-qPCR assays of selected mRNA targets.

### Statistical analyses

Paired-end reads (2 X 100 nucleotides) for the four citrus cDNA libraries were generated with the Illumina HiSeq 2500 and mapped to *Citrus sinensis* ‘Valencia’ reference genome [13] with Bowtie (http://bowtie-bio.sourceforge.net/index.shtml). Differentially expressed transcripts (DETs) were identified for CTV-B2, CTV-B6 and CaLas-B232 infected citrus libraries against the mock-inoculated library with the Gfold program [114]. Gfold is especially useful when replicate data is not available. Gfold normalizes the expression level of genes on reads per kilobase per million reads (RPKM) and tests for significance with the Poisson distribution. *P* < 0.01 and log_2_ fold change (log_2_FC) ≥ │1│were set as cut-off values. DETs were assigned into GO (Gene Ontology) categories, including biological processes (BPs), molecular functions (MFs) and cellular components (CCs) based on *A. thaliana* orthologs by PlantGSEA [115] (Gene Set Enrichment Analysis) [115] with a hypergeometric test (*p*-values < 0.05). Functional annotation of DETs was also performed with Mapman [116] based on *Citrus sinensis* orthologs. The raw RNA-Seq data has been deposited at the sequence read archive of NCBI under the project number SRP067360.

### RT-qPCR analysis

A total of 38 genes were assayed by RT-qPCR with RNA preparations from true biological replicates to validate the Gfold results. Genes were selected based on their predicted function in disease symptom development and fold changes (log_2_FC) estimated by Gfold. Gene-specific primers were designed with calculated melting temperatures of 60 °C ± 5 °C by primer (Additional file 1: Table S11; Integrated DNA Technologies, Coralville, IA). Melting curve analysis was performed to ensure that a single product was amplified. The 2^-△△Ct^ method was applied for relative quantification of gene expression [117]. The correlation between Gfold and RT-qPCR was performed with SPSS 16.0.

## Additional file

Additional file 1: Figure S1. Summary of reads from *Citrus sinensis* infected with CTV-B2, CTV-B6 and CaLas-B232 and the mock-inoculated control (H). Reads were obtained through paired-end RNA sequencing and mapped to the *C. sinensis* genome. **Figure S2**. Differentially expressed transcripts in diseased compared to healthy *C. sinensis*. Transcripts were up- or down-regulated in response to infection by CTV-B2, CTV-B6 and CaLas-B232. **Figure S3.** Melt curves of gene products assayed by RT-qPCR demonstrate specificity and reproducibility of the qPCR reactions. Gene targets are described in Table S11. **Figure S4**. Summary of differentially expressed transcripts based on functional categorization by Mapman. **A**. cell wall modification and cell organization; **B**. transcription factors; **C**. transporters; **D**. hormone metabolism; **E**. secondary metabolism; **F**. signaling; **G**. disease resistance proteins; **H**. proteolysis; **I**. development. **Figure S5**. Leaves that were sampled for RNA-seq. **A**, healthy; **B**, CTV-B2 positive; **C**, CTV-B6 positive; **D**, CaLas-B232 positive. **Table S1**. Differentially expressed transcripts of genes with unknown functions in response to CTV-B2, CTV-B6 and CaLas-B232. **Table S2**. GO categorization of genes that were differentially expressed in response to CTV-B2, CTV-B6 and CaLas-B232. **Table S3**. GO categorization of genes that were differentially expressed in response to CTV-B2, CTV-B6 but not CaLas-B232. **Table S4**. GO categorization of genes that responded to CTV-B2 and CaLas-B232 but not CTV-B6. **Table S5**. GO categorization of genes that responded to CTV-B6 and CaLas-B232 but not CTV-B2. **Table S6**. GO categorization of genes that responded only to CTV-B2. **Table S**7. GO categorization of genes that responded only to CTV-B6. **Table S8**. GO categorization of genes that responded only to CaLas-B232. **Table S9**. Complete set of differentially expressed transcripts in response to the three pathogens. **Table S10**. Estimates of changes in gene expression (fold change) by RT-qPCR and Gfold. **Table S11**. Primers used for qRT-PCR validation of differentially expressed transcripts identified by Gfold. (ZIP 1765 kb)

## Abbreviations

BP: biological process
CaLas: ‘*Candidatus* Liberibacter asiaticus’
CC: cellular component
CTV: *Citrus tristeza virus*
DET: differentially expressed transcript
FC: fold change
GO: Gene Ontology
GSEA: Gene Set Enrichment Analysis
HLB: huanglongbing
MF: molecular function
RPKM: Reads per KB per million
TF: transcription factor
