# Supplementary material for: Transcriptome analysis of sweet orange trees infected with ‘Candidatus Liberibacter asiaticus’ and two strains of Citrus Tristeza Virus
Source: BMC Genomics. 2016 May 11;17:349. doi: 10.1186/s12864-016-2663-9 (PMC4865098; doi:10.1186/s12864-016-2663-9)
Supplement: Additional file 1: Figure S1. — Summary of reads from Citrus sinensis infected with CTV-B2, CTV-B6 and CaLas-B232 and the mock-inoculated control (H). Reads were obtained through paired-end RNA sequencing and mapped to the C. sinensis genome. Figure S2. Differentially expressed transcripts in diseased compared to healthy C. sinensis. Transcripts were up- or down-regulated in response to infection by CTV-B2, CTV-B6 and CaLas-B232. Figure S3. Melt curves of gene products assayed by RT-qPCR demonstrate specificity and reproducibility of the qPCR reactions. Gene targets are described in Table S11. Figure S4. Summary of differentially expressed transcripts based on functional categorization by Mapman. A. cell wall modification and cell organization; B. transcription factors; C. transporters; D. hormone metabolism; E. secondary metabolism; F. signaling; G. disease resistance proteins; H. proteolysis; I. development. Figure S5. Leaves that were sampled for RNA-seq. A, healthy; B, CTV-B2 positive; C, CTV-B6 positive; D, CaLas-B232 positive. Table S1. Differentially expressed transcripts of genes with unknown functions in response to CTV-B2, CTV-B6 and CaLas-B232. Table S2. GO categorization of genes that were differentially expressed in response to CTV-B2, CTV-B6 and CaLas-B232. Table S3. GO categorization of genes that were differentially expressed in response to CTV-B2, CTV-B6 but not CaLas-B232. Table S4. GO categorization of genes that responded to CTV-B2 and CaLas-B232 but not CTV-B6. Table S5. GO categorization of genes that responded to CTV-B6 and CaLas-B232 but not CTV-B2. Table S6. GO categorization of genes that responded only to CTV-B2. Table S7. GO categorization of genes that responded only to CTV-B6. Table S8. GO categorization of genes that responded only to CaLas-B232. Table S9. Complete set of differentially expressed transcripts in response to the three pathogens. Table S10. Estimates of changes in gene expression (fold change) by RT-qPCR and Gfold. Table S11. Primers used for qRT-PCR va [file 12864_2016_2663_MOESM1_ESM.zip › Additional file 1/Figure S1.pdf]

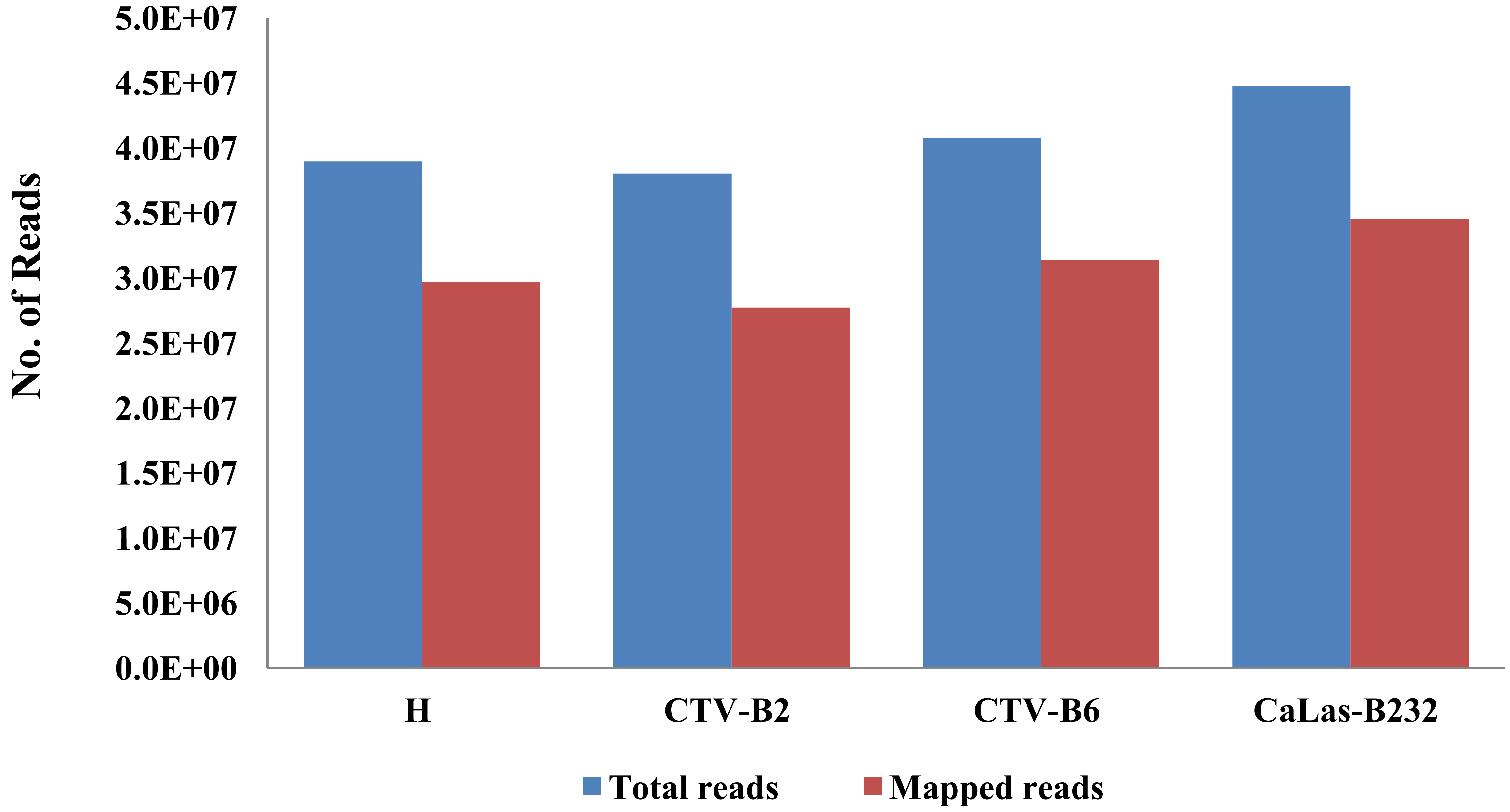

Figure S1. Summary of reads from *Citrus sinensis* and that were infected with CTV-B2, CTV-B6 and CaLas-B232 and the mock-inoculated control (H). Reads were obtained through paired-end RNA sequencing and mapped to the *C. sinensis* genome.
